# A Loss of Function Analysis of Host Factors Influencing *Vaccinia virus* Replication by RNA Interference

**DOI:** 10.1371/journal.pone.0098431

**Published:** 2014-06-05

**Authors:** Philippa M. Beard, Samantha J. Griffiths, Orland Gonzalez, Ismar R. Haga, Tali Pechenick Jowers, Danielle K. Reynolds, Jan Wildenhain, Hille Tekotte, Manfred Auer, Mike Tyers, Peter Ghazal, Ralf Zimmer, Jürgen Haas

**Affiliations:** 1 The Roslin Institute and Royal (Dick) School of Veterinary Studies, University of Edinburgh, Edinburgh, Midlothian, United Kingdom; 2 Division of Pathway Medicine, University of Edinburgh, Edinburgh, Midlothian, United Kingdom; 3 Institute for Informatics, Ludwig-Maximilians-Universität München, München, Germany; 4 Wellcome Trust Centre for Cell Biology, University of Edinburgh, Kings Buildings, Edinburgh, Midlothian, United Kingdom; 5 Institute for Research in Immunology and Cancer, Universite de Montreal, Montreal, Quebec, Canada; 6 SynthSys, Centre for Integrative Systems at Edinburgh, University of Edinburgh, Edinburgh, Midlothian, United Kingdom; University of Liverpool, United Kingdom

## Abstract

*Vaccinia virus* (VACV) is a large, cytoplasmic, double-stranded DNA virus that requires complex interactions with host proteins in order to replicate. To explore these interactions a functional high throughput small interfering RNA (siRNA) screen targeting 6719 druggable cellular genes was undertaken to identify host factors (HF) influencing the replication and spread of an eGFP-tagged VACV. The experimental design incorporated a low multiplicity of infection, thereby enhancing detection of cellular proteins involved in cell-to-cell spread of VACV. The screen revealed 153 pro- and 149 anti-viral HFs that strongly influenced VACV replication. These HFs were investigated further by comparisons with transcriptional profiling data sets and HFs identified in RNAi screens of other viruses. In addition, functional and pathway analysis of the entire screen was carried out to highlight cellular mechanisms involved in VACV replication. This revealed, as anticipated, that many pro-viral HFs are involved in translation of mRNA and, unexpectedly, suggested that a range of proteins involved in cellular transcriptional processes and several DNA repair pathways possess anti-viral activity. Multiple components of the AMPK complex were found to act as pro-viral HFs, while several septins, a group of highly conserved GTP binding proteins with a role in sequestering intracellular bacteria, were identified as strong anti-viral VACV HFs. This screen has identified novel and previously unexplored roles for cellular factors in poxvirus replication. This advancement in our understanding of the VACV life cycle provides a reliable knowledge base for the improvement of poxvirus-based vaccine vectors and development of anti-viral theraputics.

## Introduction

*Vaccinia virus* (VACV) is a large double-stranded DNA virus with a complex cytoplasmic life cycle. It is the prototypical member of the orthopoxviridae genus of the Poxviridae family which includes *Variola virus* (the causative agent of smallpox), *Monkeypox virus* and *Ectromelia virus*. VACV was used as a vaccine in the successful global eradication of smallpox in the 20^th^ century and closely related attenuated strains such as Modified Vaccinia virus Ankara (MVA) are now some of the most frequently used recombinant vaccine vectors against a variety of human and animal diseases including HIV, malaria and tuberculosis [1]. Understanding the VACV life cycle is therefore important since it provides the base for the development of efficient and safe novel vaccines.

VACV, like all other viruses, harnesses the cell to enable its replication. It turns off or subverts multiple crucial anti-viral pathways including cytokine production, Toll-like receptor pathways, NF-κB activation and the dsRNA PKR response [2]–[8]. In addition VACV suppresses both intrinsic and extrinsic pro-apoptotic pathways [9] and activates numerous anti-apoptotic, pro-survival pathways including the PI3K/Akt pathway [10], [11], the MEK/ERK pathway [12], [13], the p38 MAPK pathway [14] and the MAPK/JNK pathway [14], [15]. Modulation of so many different signalling pathways prevents viral-induced premature cell death and contributes to the ability of poxviruses to replicate in a wide range of cell types.

To investigate this complex pathogen-host relationship further, a RNAi screen of druggable host targets was carried out to analyse the effect of cellular protein depletion on VACV replication, using a multi-cycle VACV infection assay that monitors all stages of virus replication including virus spread. The screen identified a range of previously identified HFs, but also novel HFs and pathways influencing VACV infection that may facilitate the development of broadly effective anti-viral strategies and the optimisation of poxviral-based vaccine vectors.

## Materials and Methods

### RNA Interference Screen

**Figure 1:**
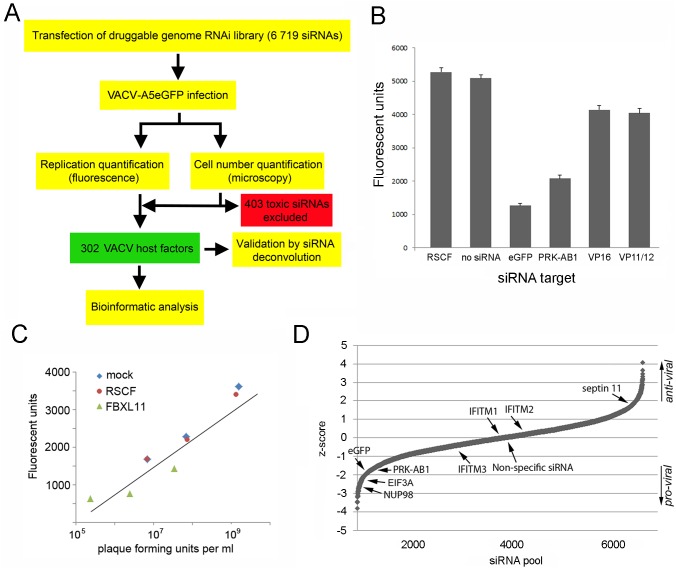
Identification of HFs for *Vaccinia virus* replication by RNA interference screen. (a) Schematic of the experimental workflow used to screen the replication of VACV with the druggable RNAi library. (b) Comparison of the level of fluorescence of the control siRNAs used in the primary screen. Wells were transfected with siRNA targeting PRK-AB1 and eGFP (known to downregulate VACV-A5eGFP growth), two negative controls (mock transfection and RSCF siRNA) and two non-specific siRNAs (targeting VP16 or VP11/12 from *Herpes simplex virus* type 1). Error bars indicate the standard error of the mean. (c) Correlation between level of fluorescence and amount of virus present. HeLa cells were mock transfected or transfected with siRNA which is not processed by the RISC machinery (RSCF) or which knocks down a strong VACV pro-viral factor (FBXL11). After 48 h cells were infected with VACV-A5eGFP at low multiplicity of infection (MOI 0.05). At 24, 36 and 48 h post infection fluorescence was measured (y axis) before the cells were collected for titration using a plaque assay (x axis). Correlation (Pearson product moment correlation coefficient) between the two datasets = 0.86. (d) Plot of sorted z-scores representing the level of fluorescence associated with each of the 6 719 siRNA SMARTpools in the screen (average of 8 replicates). siRNA pools targeting genes of particular interest are marked.

A schematic diagram of the workflow used in the RNAi screen is shown in **Figure 1**. siRNA SMARTpools (4 siRNAs per gene, Dharmacon) were diluted to 0.3 µM and dispensed in 10 µl volumes using a Rapidplate384 liquid handler (Qiagen) into eight black 384-well plates (Corning). These were stored at −80°C until needed (maximum 48 h). On the day of transfection, plates were thawed and 10 µl transfection reagent (Dharmafect 1, Dharmacon) diluted in Hank’s buffered saline solution (HBSS, ThermoFisher) was added to each well containing siRNA using a Multidrop 384 (ThermoFisher), to give a final transfection reagent concentration of 0.1%. Plates were incubated for 20 min at room temperature to allow formation of transfection complexes. During complex formation, low-passage (p20–22) HeLa cells (ECACC) from approximately 50% confluent flasks were washed in PBS and trypsinised in Trypsin-EDTA (Lonza) before diluting in phenol red-free, antibiotic-free transfection medium (DMEM/F-12 1∶1 with 5% FCS, 15 mM Hepes and L-glu; Gibco). 3×10^3^ cells in a volume of 40 µl were added to each well using the Multidrop 384. Plates were incubated for 48 h at 37°C in a humidified incubator with 5% CO_2_ before infection. To infect, media was removed from plates by inversion, and 15 µl media (DMEM +4.5 g/L D-glucose, L-glu and pyruvate with 2.5% FCS and penicillin-streptomycin) or 15 µl media containing VACV strain WR with eGFP tagged A5 protein [16] diluted to MOI 0.05, was added using the Multidrop 384. Plates were incubated at 37°C for 1 h before 50 µl of media was added to each well, the plates inverted to remove the media and virus, and a final volume of 50 µl of media added to the plates before they were returned to the incubator. After 48 h the plates were inverted to remove the media and 50 µl of 10% buffered formal saline added to fix the cells. Fluorescence levels were measured using a POLARstar OPTIMA plate reader (BMG Labtech). Data from eight replicates was used for analysis. Background intensity correction was carried out by subtracting the median value of uninfected wells and the data was normalised using the robust Z score method [17], and corrected for the number of cells in each well. The correction for the number of cells in each well was carried out by estimating the linear correlation coefficient between the level of fluorescence (phenotype score) and the number of cells (toxicity score) using least squares optimization. This coefficient was used to linearly adjust the phenotype scores.

### High Content Screening

One replicate of the screen was imaged by a high content screening system. The buffered formal saline was removed from the cells by inverting the plates, and cells were washed in 50 µl of room temperature PBS before permeabilising for 15 min at room temperature in 30 µl of 0.1% tritonX-100 diluted in PBS. Plates were inverted and 50 µl of a 1∶50 dilution of AlexaFluor-647 phalloidin (Invitrogen Molecular Probes) diluted in PBS + 1% BSA was added and incubated for 45 min in the dark. The phalloidin was removed by inversion and 50 µl of DAPI (1 µg/ml) diluted in PBS was added and left on. Cells were analysed by automated microscopy using an OPERA high content screening system (Perkin Elmer) and Acapella High Content Imaging and Analysis software.

### Definition of Toxic siRNA Pools

To identify siRNA SMARTpools which exerted significantly toxic effects the number of cells in each well was counted and converted to a z-score. A z-score is equivalent to the number of standard deviations away from the mean. siRNA treatments that reduced the cell number by two or more standard deviations below the population mean (z-score of −2 or less) were removed from further analysis. A z-score of −2 was equivalent to 250 cells, compared to a population mean of 455.

### qPCR Confirmation of siRNA Knockdown

Selected siRNA SMARTpools were diluted to 0.3 µM in 1x siRNA buffer and dispensed in triplicate in 96-well plates (Corning). To this, 10 µl Dharmafect 1 diluted in DMEM to give a final concentration of 0.15% was added using the Multidrop 384. Following a 20 min incubation to enable complex formation, 0.4×10^4^ Hela cells in 80 µl transfection media were added and plates were transferred to a 37°C humidified incubator with 5% CO_2_. After 48 h, medium was removed and cells rinsed in PBS before lysing in 100 µl TRIZOL (Invitrogen). Triplicate wells were combined, and RNA extracted by PureLink (TM) RNA Mini Kit (Life Technologies). mRNA levels were determined by either TaqMan qPCR with gene-specific primers and probes from the Universal Probe Library (Roche), or by SYBR green qPCR, using the appropriate one-step RT-qPCR kits (Thermofisher). Expression levels were normalised to the housekeeping cellular gene hypoxanthine phosphoribosyltransferase 1 (HPRT) and calibrated to mock-transfected cells. qPCR was carried out in duplicate for each sample, and normalised expression levels averaged.

### Phenotype Validation by siRNA Deconvolution

The phenotype observed in the primary screen was confirmed for a subset of candidate genes with deconvoluted siRNA SMARTpools. The four individual siRNAs targeting different regions of each gene were diluted to 0.3 µM in 1x siRNA buffer and dispensed to 96-well plates in triplicate. Transfection and, 48 h later, infection with VACV-A5eGFP was carried out as described above. At 0 and 48 h post infection fluorescence was measured using a Synergy HT plate reader (BioTek). The experiment was carried out three times to produce a dataset of three biological replicates each containing three technical replicates. The data were analysed using mixed models [18] fitting gene, time-point gene*time interaction and first time-point value as fixed effects. Values observed at the first time-point were fitted as a ‘baseline covariate’ in order to increase the sensitivity of the analysis. The repeated experiments were fitted as random effects, causing the variation in results between the repeated experiments to be taken into account when testing statistical significance. Differences between the amount of fluorescence present in wells treated with each siRNA and wells treated with a non-specific siRNA (targeting the HSV-1 gene VP16) were tested within the mixed models using t-tests. Groups of genes were tested on separate plates and each of the groups were analysed using a separate mixed model. A phenotype was considered confirmed if two or more of the four siRNAs resulted in a *p*-value of 0.05 or less.

### Plaque Assay

Six wells of a 96 well plate were transfected with a siRNA SMARTpool and, 48 h later, infected with VACV-A5eGFP as described above. At 24, 36 and 48 h post infection cells were scraped into the overlying media, collected and then frozen and thawed three times and sonicated for 30 seconds (Misonix sonicator 3000). The resultant lysate was titrated on BS-C-1 cell monolayers and virus titre quantified as plaque forming units (PFU) per ml [19].

### Gene Set Overrepresentation Analysis

Enrichment analysis was performed with respect to pathway- and GO-based gene sets defined in MSigDB [20], as well as with respect to gene sets derived from protein complexes curated in the CORUM [21] and PIN [22] databases. Specifically, the genes were sorted based on the screening data, and then the propensity of gene sets towards pro- or anti-viral activities were sought out using rank-sum tests with multiple testing [23].

### Comparison with Cellular Expression Data

The three gene expression data sets used in the analyses were data set A (GSE11238, a microarray of VACV infected HeLa cells (http://www.be-md.ncbi.nlm.nih.gov/projects/geo/query/acc.cgi?acc=GSE11238)), data set B (GSE24125, a microarray of macrophages, monocytes and fibroblasts [24]), and data set C (SRA017695, a RNA-seq based analysis of gene expression in VACV infected HeLa cells [25]). For comparison of the RNAi hit list with the two HeLa cell based cellular expression data sets, the differential expression-based rank for each gene (g) was obtained in each condition (i.e. a timepoint in a data set) and then a “summerisation q-value” q_g_ was calculated which reflects how unexpectedly high the ranks are (using order statistics; in particular the rank of the third 4-quantile). Therefore q_g_ quantifies how unexpectedly often g is among the most strongly regulated genes (up or down).

## Results and Discussion

### Identification of *Vaccinia virus* Host Factors by RNA Interference Screen

To identify HFs that influence VACV replication we used a druggable genome small interfering RNA (siRNA) library (Dharmacon) in a high-throughput screen. This library targets genes that are considered potential candidates for therapeutics (**Figure 1a**). Briefly, SMARTpool siRNAs (a mix of 4 siRNAs per gene) targeting 6 719 genes were distributed into 384-well plates and reverse-transfected into HeLa cells. Cells were infected 48 h post-transfection at a low multiplicity of infection (MOI 0.05) with the VACV strain VACV-A5eGFP. After 48 h (thus allowing multiple complete virus replication cycles), eGFP fluorescence was quantified as a measure of infection and compared to controls in order to determine the effect of individual gene depletion on VACV replication. Two positive siRNA controls known to downregulate VACV-A5eGFP growth (targeting PRK-AB1 and eGFP), two negative controls (mock transfection and RSCF siRNA which is not processed by the RISC machinery) and two non-specific siRNAs (targeting VP16 or VP11/12 from *Herpes simplex virus* type 1) were included in duplicate in each plate (**Figure 1b**). To confirm that the measurement of virus-expressed fluorescence was a reliable marker of viral replication, fluorescence was correlated to virus-titre, as determined by standard plaque assay, over a range of time-points post-infection after treatment with control or inhibitory siRNAs (**Figure 1c**). This resulted in a Pearson product moment correlation coefficient of 0.86, confirming that fluorescence was a reliable determinant of virus replication. The entire druggable screen was repeated four times in duplicate to generate a robust primary data set of eight replicates. Pairwise agreement comparing the levels of fluorescence across the eight replicates revealed good reproducibility (median Spearman’s coefficient 0.55). One replicate of the VACV-infected cells was analysed by automated microscopy using an OPERA high content screening system and Acapella High Content Imaging and Analysis software to quantify the number of cells present in each well. A total of 403 siRNA pools (6% of the total) were associated with a significant reduction in cell number (**Figure 1a**). These were removed from further analysis and are listed in **Table S1 in File S1**.

The fluorescence data from the remaining wells in the primary screen was normalised platewise using the robust z-score method [17]. A summary value was calculated for each gene by taking the mean across the replicates, and these values were converted to z-scores which were corrected for the number of cells in the well to produce the level of fluorescence per cell for each siRNA. A negative z-score indicated a reduction in VACV replication and a positive z-score indicated an increase in VACV replication (**Figure 1d**). The two positive controls (siRNA targeting PRKAB1 and eGFP) produced strongly negative z-scores as expected. The median level of fluorescence (z-score of 0) was very close to the level of fluorescence seen in wells transfected with the non-specific siRNA (negative control), indicating that roughly half of the siRNA pools caused an increase in fluorescence and half caused a decrease. A “hit” was defined as a siRNA pool which generated a z-score of ≥2 or ≤–2. Using these criteria, a hitlist of 302 HFs (4.5% of the total) was generated, consisting of 153 pro-viral HFs which inhibited replication upon depletion and 149 anti-viral HFs which increased replication upon depletion (**Table S2 in File S1**).

### Validation of Primary RNAi Screen Data

To confirm the effect of the siRNA SMARTpools on mRNA levels, a subset of SMARTpools were transfected into HeLa cells and after 48 h, total RNA extracted and subjected to a quantitative RT-PCR to determine the level of mRNA of the targeted gene. 62 genes out of 80 tested (78%) had their transcript level reduced by 50% or more, indicating that the majority of the SMARTpools functioned as expected (**Table S3 in File S1**).

To confirm the effect of gene depletion on VACV replication a subset of HFs, chosen on the basis of their potential for further investigation, were tested using the four individual, deconvoluted siRNAs of each SMARTpool. The level of viral fluorescence was compared to that seen with non-specific siRNA and a statistically significant reduction or increase in fluorescence (p<0.05) induced by at least 2 individual siRNAs from the original SMARTpool was required for confirmation of the hit. Overall 38 (53%) out of 72 candidate genes tested by this method were successfully confirmed (**Table S4 in File S1**). This level of validation is commensurate with similar RNAi screens of viral replication which have reported confirmation of between 38% and 83% of primary screen hits [26]–[33].

**Figure 2:**
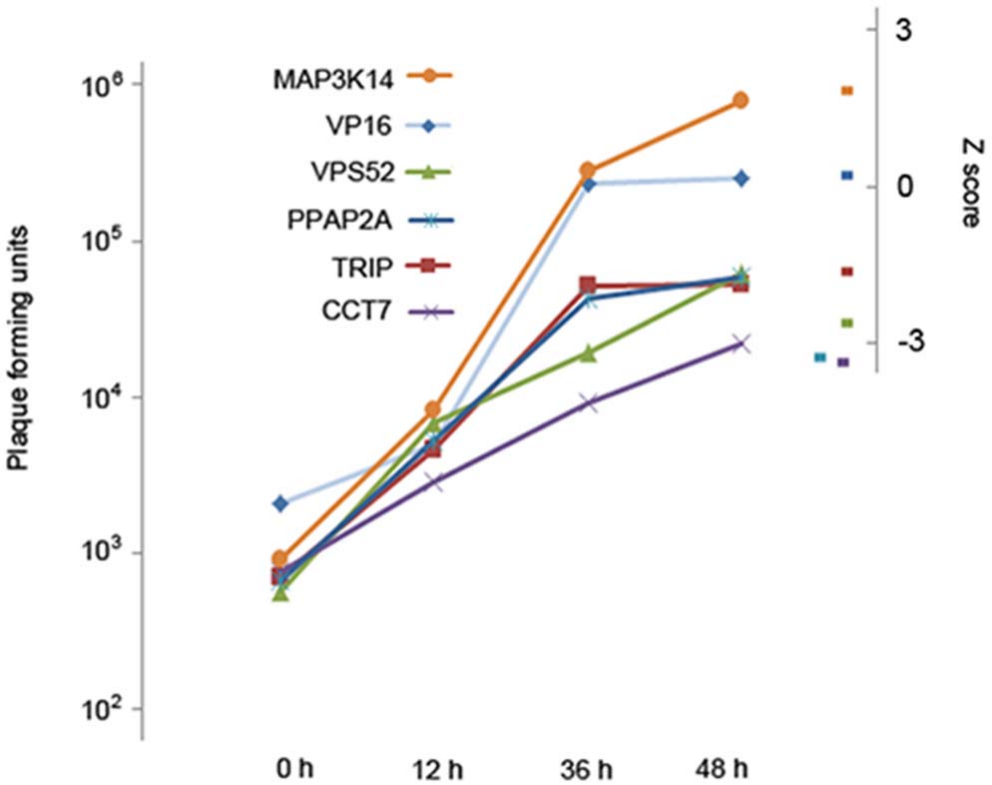
Validation of *Vaccinia virus* HFs. (a) Validation of primary screen hits using plaque assays. siRNA SMARTpools targeting five genes identified in the primary RNAi screen as modulating VACV growth (one anti-viral factor MAP3K14 and four pro-viral factors TRIP, PPAP2A, VPS52 and CCT7), and one non-specific SMARTpool (VP16) were transfected into HeLa cells and, after 48 h, infected at low MOI (0.05) with VACV-A5eGFP. At 12 h intervals, cells were collected and the amount of virus present calculated using a plaque assay. Results obtained in the primary RNAi screen are plotted on the right hand axis for comparison.

Within our deconvoluted dataset the validation level of putative pro-viral HFs was notably higher than that for the anti-viral HFs. Only 30% of the anti-viral hits (7/24) were successfully validated in comparison to 69% of the pro-viral hits (24/35) and 54% of the siRNA pools with no effect in the primary screen (7/13). One potential reason for the lower validation rate of the anti-viral hits might be that the dynamic range of the virus replication assay is such that inhibitory effects are more easily demonstrated, as under normal replication conditions virtually all cells in the well became infected by 48 h post infection (data not shown) suggesting the system reached near saturation. To examine this further, the effect of selected siRNAs on traditional viral growth curves was tested and correlated with their perturbation of fluorescence in the primary screen. HeLa cells were transfected with each one of 6 siRNA SMARTpools (upregulatory MAP3K14, control VP16, and downregulatory TRIP, PPAP2A, VPS52 and CCT7) and infected with VACV-A5eGFP at an MOI of 0.05 after 48 h. Analysis of virus titre at 12 h intervals by plaque assay found the endpoint titres correlated very closely with the z-score obtained from the primary RNAi screen (**Figure 2**), further validating the robustness of the primary screen. Notably, however, whilst the inhibitory siRNAs decreased the maximum virus titre by between 13- (CCT7) and 5-fold (VPS52) the siRNA treatment directed against a candidate antiviral HF (MAP3K14) only increased peak virus titre by 3-fold. Thus the response of the system is more suited to detecting inhibitory perturbation, with relatively reduced sensitivity for detection of individual anti-viral HFs.

The list of 302 individual HFs was analysed for genes already known to influence VACV replication. Numerous examples were found including clathrin and proteins involved in Golgi vesicular trafficking, both of which are required for the production of enveloped VACV forms [34], [35], as well as multiple components of the AMPK complex which has been shown to aid VACV entry [36]. In addition the signalling pathway regulating protein TRAF2 was identified in the screen as a pro-viral hit; further work demonstrated that it promoted rapid VACV entry [37]. The identification of known host factors for VACV and our follow-up identification of the role of TRAF2 in VACV replication supports the reliability and significance of this RNAi screen dataset.

Overall, eight replicates of a genome-wide RNAi screen of multiple VACV replication cycles identified 302 cellular genes, consisting of 153 HFs that positively support VACV replication and 149 HF with anti-viral effects.

### Host Factors Common to other VACV Screens

**Figure 3:**
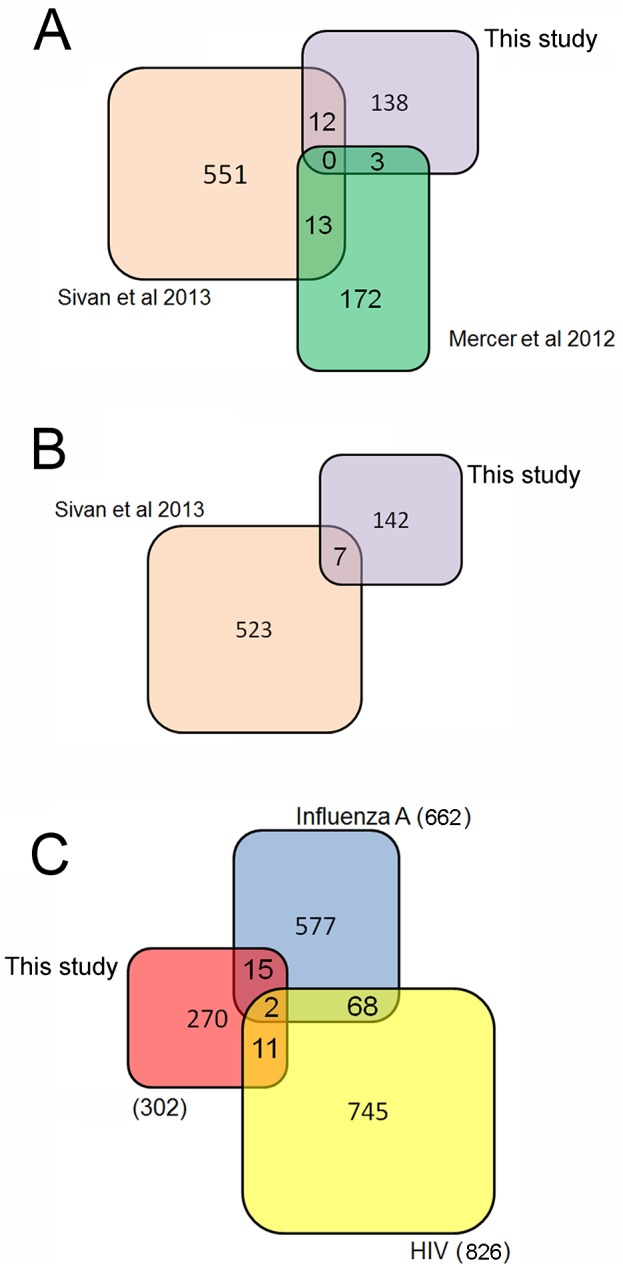
Identification of anti and pro-viral HFs common to multiple RNAi viral screens. Venn diagram showing the (a) pro-viral and (b) anti-viral hits common to at least two VACV RNAi screens and (c) hits common to the VACV screen reported in this study and three published influenza A RNAi screens with a total of 662 hits [26], [31], [47] and three published HIV RNAi screens with a total of 826 hits [41]–[43].

To prioritise investigations of the 302 potential VACV HFs, the candidate genes were compared to the hit lists of other viral RNAi screens, including two recently published VACV screens [32], [38]. The methodology in the previously published VACV screens varied considerably; Mercer et al [32] measured the growth of a thymidine-kinase-deficient VACV (strain Western Reserve) after only 8 h of infection, thereby identifying cellular proteins involved in the initial stages of virus replication but excluding analysis of viral spread. They reported 188 pro-viral HF but no anti-viral HFs. A second screen by Sivan et al [38] used the VACV strain IHD-J (which has a point mutation that accelerates the release of progeny virions from the cell surface) to identify genes which influenced viral replication after 18 h of infection, thus measuring the entire replication cycle with emphasis on viral spread. They reported 576 pro-viral and 530 anti-viral HFs. The overlap between the hit lists reported by the three VACV RNAi studies (Mercer et al, Sivan et al, and this study) is depicted in **Figure 3a and b** and the HFs common to two studies are listed in **Table S5 in File S1**. The number of overlapping hits between two of the screens ranged from 3 to 13 and no HFs were common to all three VACV studies. A small number of common hits between siRNA screens of the same virus is a frequent finding [39], [40] and, given the variation in methodology between the three VACV screens (including viral strain, infection time, and data analysis), is not surprising. However, comparison of the enriched functions and pathways identified in each of the three VACV screens revealed marked similarities (discussed below), demonstrating the power of comparative screening approaches to identify significant cellular pathways involved in virus replication.

### Host Factors Common with other Viruses

Genome-scale siRNA screens have been carried out for many viruses other than VACV, including HIV-1 [41]–[43], West Nile Virus (WNV) [44], Hepatitis C Virus (HCV) [30], [45], Vesicular Stomatitis Virus (VSV) [29], Borna Disease Virus [46], enteroviruses [27], Dengue virus [28], herpes simplex virus 1 (HSV-1) [33] and influenza A virus [26], [31], [47]. Host factors common to two or more of these screens could represent broadly acting cellular proteins with a generalised effect on viral replication. A comparison of the VACV HFs identified in this screen with those identified in other viral screens found a small overlap with WNV, VSV, Borna Disease virus and Dengue virus, whilst 21 VACV HFs were shared with HSV-1, 17 with influenza A virus and 13 with HIV-1 (**Figure 3c**). A list of overlapping genes can be found in **Table S6 in File S1**.

Amongst the factors in common, the nucleocytoplasmic transport factor NUP98 was identified as a proviral hit in the VACV screen reported here as well as HIV, HSV-1 and influenza A virus screens [26], [31], [33], [42]. It is located at both the cytoplasmic and the nuclear faces of the central channel of the nuclear pore complex (NPC) [48], is involved in Rev-dependent RNA export during HIV infection [49], and has been shown to play both pro- and anti-viral functions during influenza A virus infection [31], [50], [51]. NUP98 was a somewhat unexpected proviral hit in our screen since poxvirus replication and assembly occur in the cytoplasm. However the two VACV RNAi screens published recently also identified a number of nuclear pore proteins as pro-viral, with one screen demonstrating that knockdown of NUP62 strongly inhibited viral morphogenesis [38]. The number of nuclear pore proteins now identified as pro-viral HFs strongly suggests poxviruses require functional nuclear membrane transport for efficient replication.

Another HF that affects both VACV and influenza A virus replication is MAP2K3 (also known as MKK3 and MEK3), which activates the p38 MAPK signalling pathway and is involved in low pH-dependent entry of influenza virus and VSV [31], [47]. VACV also has a low pH-dependent entry mechanism [52] which may be similarly reliant on MAP2K3. Alternatively, it may be required to activate the p38 MAPK pathway to promote cell survival post infection [14].

In contrast, IFITMs (interferon inducible transmembrane proteins) have been identified in functional genomic screens as mediating resistance to influenza A virus, Dengue virus and West Nile virus infection *in vitro* and *in vivo* [26], [53] as well as Marburg and Ebola viruses, SARS-coronavirus [54] and HIV [55]. These proteins prevent entry of viruses at the plasma membrane, endosomes and lysosomes [56], however none had an effect on VACV replication in our screen, suggesting VACV is resistant to the repressive effect of this protein family (**Figure 1d**).

### Transcriptional Modulation of *Vaccinia virus* Host Factors

**Figure 4:**
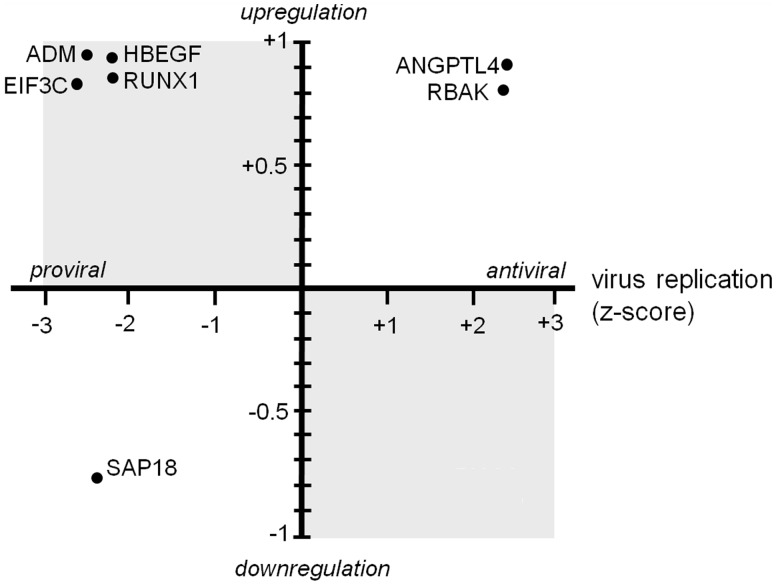
Transcriptional modulation of *Vaccinia virus* HFs. Plot of seven VACV HFs identified in the RNAi screen that are also strongly transcriptionally regulated in VACV infected cells. The x-axis represents the level of fluorescence in the RNAi screen (viral replication) expressed as a z-score with pro-viral genes to the left and anti-viral genes to the right. The y-axis represents the relative expression of the seven genes in VACV infected cells.

To determine whether the expression of HFs identified by RNAi is modulated by VACV, the RNAi hit list was compared to previously published transcriptional profiling data sets performed on cells infected with VACV. Three available expression profiling data sets (designated A, B and C, see Materials and Methods for sources) which used different cells, virus isolates and time points were compiled and compared. Correlation was mainly limited to intra-data set measurements (i.e. different time points or cell lines within a data set) with some examples of agreement between data sets A and C, both of which used HeLa cells. To test whether there was a general propensity of HFs to be differentially expressed in virus-infected cells the distribution of the 302 RNAi hits was compared to that of the whole RNAi screen in these three transcriptional profiling data sets. No significant tendency of pro-viral HFs to be up-regulated or anti-viral HFs to be downregulated was detected (data not shown). Subsequently the expression of individual HFs was examined using the transcriptional profiling data of the two HeLa-based studies (A and C). This revealed specific examples of HFs which are differentially expressed in virus-infected cells (**Figure 4**). Four pro-viral HFs (RUNX1, eIF3C, HBEGF, and ADM) were significantly upregulated in VACV-infected HeLa cells (*q*<0.05), suggesting that VACV might promote expression of these proteins to assist viral replication and spread. RUNX1 is a subunit of the transcription factor CBF which regulates critical processes in both myeloid and lymphatic haematopoiesis. Chromosomal translocations and mutations of RUNX1 are among the most frequent genomic abnormalities in different types of leukaemia [57]. Furthermore, a genome-wide association study found a genetic polymorphism in RUNX1 to be associated with the serological response to VACV vaccination (Dryvax vaccine, Wyeth Laboratories) in African-Americans. This suggests that the RUNX1 polymorphism may influence replication and viral gene expression of the live-attenuated vaccine *in vivo* which causes differences in the strength of the adaptive immune response [58], a hypothesis supported by our identification of RUNX1 as a pro-viral VACV HF.

In addition to upregulated pro-viral HFs, opposite examples were also identified, with two anti-viral HFs (ANGPTL4, RBAK) significantly upregulated and one pro-viral HF (SAP18) downregulated in VACV-infected cells. This lack of correlation between functional HFs and gene expression at the transcriptional level serves to underscore the complexity of virus-host interactions and highlights the need for further follow-up studies.

### Functional and Pathway Analyses of *Vaccinia Virus* Host Factors

**Figure 5:**
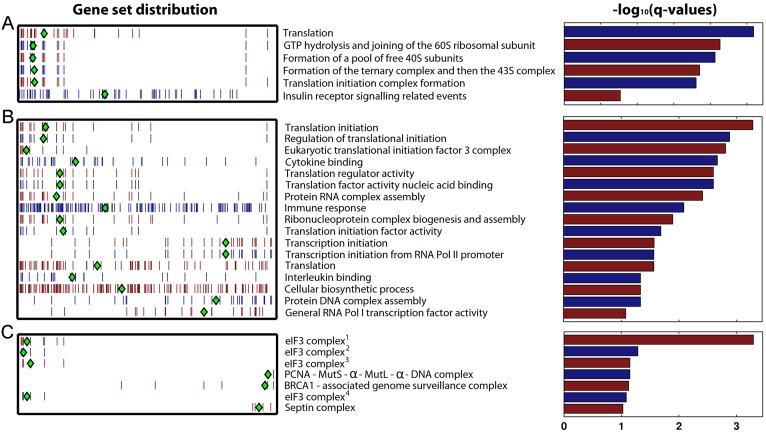
Functional characterization of *Vaccinia virus* HFs. Gene sets identified by over-representation analysis. Gene sets were identified using (a) pathway- and (b) GO-based gene sets as defined in the MSigDB database, or (c) protein complexes defined in the CORUM [21] and PIN [22] databases. All significantly overrepresented gene sets (–log_10_(q-value)>0.1) are shown. Each row shows the ranks of genes from a particular gene set that were present in the RNAi screen. Each tick mark denotes the place of a particular gene from that gene set, placed at the appropriate position in the distribution. Genes were sorted from left to right from most pro-viral to most anti-viral. The red and blue colours of the ticks are used for visual contrast. A green diamond is used to denote the median rank of the genes in the set.

To assess further the role of candidate HFs and associated functions and pathways in VACV replication, an overrepresentation analysis of the complete VACV RNAi data set was performed with respect to pathway- (**Figure 5a**) and GO- (**Figure 5b**) based gene sets as defined in the MSigDB database, as well as from curated protein complexes defined in the CORUM [21] and PIN [22] databases (**Figure 5c**). Translation was the most strongly enriched theme in all these analyses, particularly the eIF3 complex. Poxviruses are known to utilise the host translation machinery for production of viral proteins therefore the enrichment of translation as a pro-viral theme is a validation of the screening method. The cellular translation machinery has been highlighted in other viral RNAi screens as essential for VSV [29] and hepatitis C virus [45] and HSV-1 [33].

**Figure 6:**
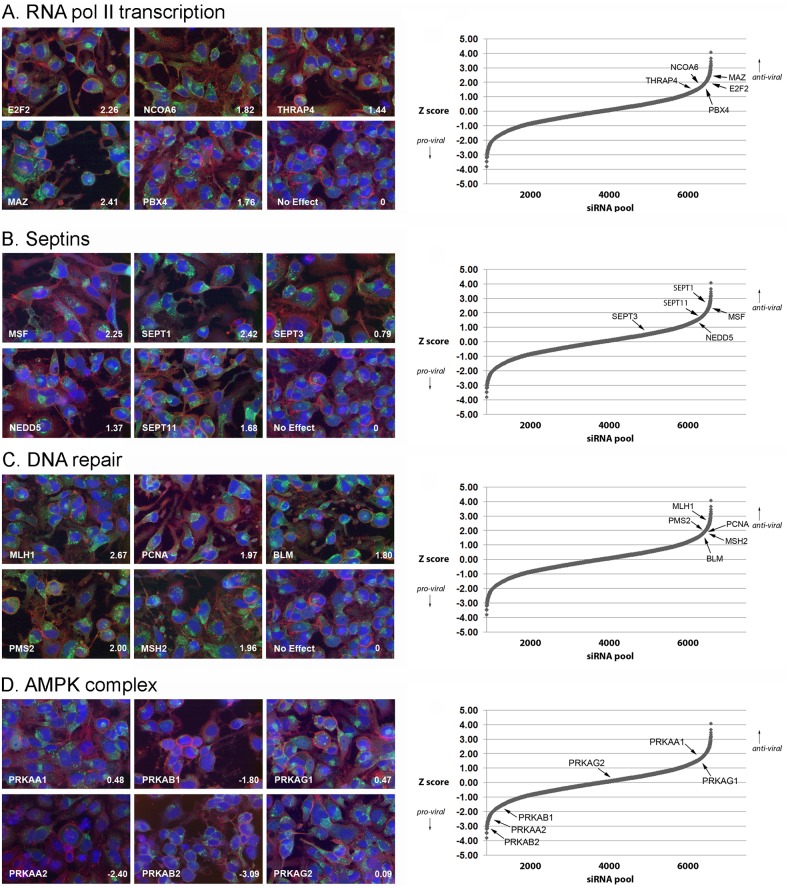
Analysis of pro- and anti-viral cellular pathways. Left hand panels show selected fluorescence images of infected HeLa cells transfected with the indicated siRNAs at 48h post infection. Blue = DAPI (DNA stain), red = phalloidin (actin cytoskeleton) and green = VACV-A5eGFP. The z-score of each siRNA is indicated in the bottom right of each image. The right hand panels show the plot of sorted z-scores from the primary screen with the position of genes of interest marked. (a) Transcriptional proteins inhibitory for VACV replication (b) Anti-viral function of septins (c) Genome maintenance and DNA repair proteins inhibitory for VACV replication (d) The AMP-activated kinase complex is involved in VACV replication.

More interestingly, transcriptional initiation and general RNA polymerase II transcription factor activity were identified in the functional analysis of the RNAi screen as significantly over-represented anti-viral GO-based gene sets (**Figure 5b**). Sevin et al [38] also reported that interference with DNA-dependent RNA polymerase II pathways enhanced VACV spread. Our work identified individual anti-viral HFs involved in transcription such as MAZ (an inflammatory responsive transcription factor also known as SAF1), and the transcription factor E2F2. Inspection of individual confocal images taken at 48 h pi of cells depleted of these factors showed notably brighter accumulations of eGFP-labelled virus, in line with the positive z-scores from the overall primary screen (**Figure 6a**). Orthopoxvirus infection results in a rapid shut down of cellular transcription, with a marked reduction in the amount of host mRNA present as early as 2 h post infection [25], [59], [60]. This effect is believed to result from cessation of host mRNA synthesis and degradation of cellular mRNA transcripts. The viral proteins involved in this shut-off of host cell transcription have not been identified, although D9 and D10 have been implicated [61], [62]. In contrast to viral translation which is dependent on host proteins, VACV encodes its own transcription enzymes so is largely unaffected by a general repression of cellular transcription [63]. Therefore VACV-induced downregulation of host transcription prevents the host cell from transcribing anti-viral, pro-inflammatory gene programmes, such as the NF-κB cascade, while having a minimal negative effect on viral transcription. Whilst VACV has developed mechanisms to shut off host transcription, these data show that virus replication is improved when transcription is impaired prior to infection. Thus, despite the efforts of the virus to shut off host transcription, some anti-viral effect of this pathway persists in VACV infected cells.

Two members of the septin protein family (septin 1 and MSF/septin 9) were identified in the RNAi screen as anti-viral HFs (**Figure 6b**). MSF/septin 9 co-purifies from cells with three other septin proteins (NEDD5/septin 2, CDC10/septin 7 and septin 11), suggesting they form a functional complex [64]. These were therefore grouped in the pathway analysis, resulting in a significant over representation (q = 0.1) (**Figure 5c**). Consistent with this, depletion of NEDD5/septin 2, septin 7 and septin 11 all increased virus replication although not to the stringent cut-off used here to define a ‘hit’ (**Figure 6b**). Deconvoluted siRNAs targeting septin 11 mRNA confirmed the enhancement of virus replication, although results with the other family members were more variable (**Table S4 in File S1**). Septin 11 has also been identified as a proviral hit in a recently reported VACV siRNA screen [38]. Septins are conserved GTP-binding proteins which act as dynamic scaffolds for recruitment of other proteins. They are involved in actin and microtubule function, cytokinesis, cell movement and vesicle trafficking [65]. Interestingly, they can be recruited together with autophagic proteins to “cage” *Shigella flexneri* in the cytosol of infected cells, restricting bacterial dissemination [66]. The cage assembly is linked with actin polymerisation activity of *S. flexneri*, suggesting that a similar mechanism may be employed by the host cell to “cage” VACV virions (which activate actin polymerisation both early and late in the replication cycle [67], [68]) and thus invoke an anti-viral effect.

Two groups of genes involved in DNA replication and repair were highlighted in the pathway analysis as having anti-viral properties (**Figure 5c**). The PCNA-MutSα-MutLα-DNA complex (PCNA, MSH2, PMS2, and MLH1) and the BRCA1-associated genome surveillance complex (RFC4, BRCA1, BLM, RFC1, MSH2 and MLH1) both promoted virus replication when individual group members were downregulated (**Figure 6c**). These pathways are both involved in DNA damage signalling and repair [69], [70], a cellular process which is targeted by numerous viruses [71]. Specifically, DNA damage signalling pathways act as a host defence mechanism in poxvirus infection, detecting and responding to foreign poxviral DNA and inducing intrinsic apoptosis [72]. The identification of the PCNA and BRCA1 gene sets as strongly anti-poxviral HFs in the RNAi screen suggests they are a part of this, or a similar, defence mechanism.

The AMP-activated kinase complex (AMPK) is a key regulator of energy metabolism. It is activated by a reduction in ATP which prompts phosphorylation of many target proteins, resulting in the activation of catabolic pathways and inhibition of anabolic pathways [73]. It has also been linked to regulation of the actin cytoskeleton [74]. AMPK is a heterotrimer comprising a catalytic α subunit and regulatory β and γ subunits. In mammals each subunit has several isoforms (PRKAA1, PRKAA2, PRKAB1, PRKAB2, PRKAG1, PRKAG2, and PRKAG3) [73]. The druggable RNAi screen reported here screened all seven genes and identified three (PRKAA2, PRKAB1, and PRKAB2) as promoters of VACV replication whose depletion led to fewer and dimmer accumulations of cytoplasmic eGFP (**Figure 6d**). This result is in broad agreement with a recently published RNAi screen of 440 cellular kinases and phosphatases in a non-permissive *Drosophila* cell model of VACV infection [36], which identified seven hits including three AMPK subunits.

## Conclusion

This study performed a loss of function analysis of HFs involved in VACV infection using RNAi. Previously identified host pathways and protein complexes which aid VACV replication, such as translation and the AMPK complex proteins, were highlighted in the RNAi screen. In addition, however, a range of novel host pathways and proteins were identified that influenced VACV infection, such as the DNA damage and repair pathways, the septin family of proteins, MAP2K3 and NUP98. As many of the genes targeted in this RNAi screen have a known drug inhibitor, this work yields a list of HFs that can potentially be targeted by novel therapeutics.

## Supporting Information

File S1 **Supporting Tables.** Table S1, List of 403 cytotoxic siRNAs which caused significant cell death. Table S2, The 302 cellular genes identified by RNAi as having a significant effect on VACV replication. Table S3, qPCR confirmation of gene depletion by siRNA SMARTpools. Table S4, Deconvolution of siRNA SMARTpools. Table S5, Overlap of HFs identified in three different VACV RNAi screens. Table S6, Overlap of HFs between VACV and other viral RNAi screens.
(XLSX)
